# Phase II study of pemetrexed and carboplatin plus bevacizumab, followed by maintenance pemetrexed and bevacizumab in Japanese patients with non-squamous non-small cell lung cancer

**DOI:** 10.3892/ol.2014.2552

**Published:** 2014-09-19

**Authors:** TAKASHI YOKOI, YOSHITARO TORII, YUICHI KATASHIBA, HIROYUKI SUGIMOTO, TSUTOMU TANIJIRI, MAKOTO OGATA, NORIKO INAGAKI, KAYOKO KIBATA, MINA HAYASHI, MAIKO NIKI, TOSHIKI SHIMIZU, TAKAYUKI MIYARA, TAKAYASU KURATA, SHOSAKU NOMURA

**Affiliations:** First Department of Internal Medicine, Kansai Medical University, Hirakata, Osaka 573-1010, Japan

**Keywords:** non-squamous non-small cell lung cancer, chemotherapy, pemetrexed, bevacizumab, Japanese

## Abstract

The present study evaluated the efficacy and safety of pemetrexed, carboplatin and bevacizumab, followed by maintenance pemetrexed and bevacizumab, in chemotherapy-naïve patients with stage IIIB/IV non-squamous non-small cell lung cancer (NSCLC). The patients were administered pemetrexed (500 mg/m^2^), carboplatin (area under the concentration-time curve, 6.0 mg/ml × min) and bevacizumab (15 mg/kg) intravenously every three weeks for up to six cycles. Patients who did not experience tumor progression remained on maintenance pemetrexed and bevacizumab until disease progression or unacceptable toxicity occurred. The primary endpoint was the overall response rate. Of the 26 patients enrolled between March 2010 and April 2011, three were excluded due to brain metastases, therefore the intention-to-treat (ITT) population consisted of 23 patients. The median age was 64 years (range, 40–74 years) and 15 patients were male. In total, six patients had a performance status of 0, and 20 had stage IV tumors. The response rate was 69.6% [95% confidence interval (CI), 47.1–86.8], the disease control rate was 100% and the time to response was 1.2 months (95% CI, 0.72–1.93). The median progression-free survival time was 8.6 months (95% CI, 5.9–10.9) and the median overall survival time was 18.6 months (95% CI, 12.9–24.8). There were no grade 3 or worse hemorrhagic events and the feasibility was modest. Overall, pemetrexed and carboplatin plus bevacizumab, followed by maintenance pemetrexed and bevacizumab, was effective and tolerable in the patients with non-squamous NSCLC, and the time to response was relatively short.

## Introduction

The leading global cause of cancer-related mortality is lung cancer (1). Overall, ~85% of patients with lung cancer have non-small cell lung cancer (NSCLC), with the majority being diagnosed with advanced-stage disease. Although NSCLC is histologically heterogeneous and can be classified into several subtypes, treatment strategies have been determined solely by the disease stage (2). Platinum-based doublet chemotherapy regimens are the standard first-line treatment for patients with advanced (stage IIIB/IV) disease, regardless of histology, as they have demonstrated a survival benefit greater than that of the best supportive care (3–5).

In phase III trials comparing the efficacy of cisplatin/pemetrexed and cisplatin/gemcitabine, overall survival (OS) was greater with cisplatin/pemetrexed in patients with adenocarcinoma and large cell carcinoma, but not in patients with squamous cell carcinoma, indicating that survival following treatment with cytotoxic agents was dependent on the histological type of the NSCLC (6). Furthermore, a large, randomized, phase III trial found that maintenance therapy with pemetrexed was effective and well-tolerated in patients with advanced non-squamous NSCLC who did not progress following induction therapy with pemetrexed plus cisplatin (7).

Molecularly-targeted agents, including those targeting epidermal growth factor, vascular endothelial growth factor (VEGF), platelet-derived growth factor and insulin-like growth factor I signaling, have been developed due to our increased understanding of the pathogenesis of NSCLC. A rationale for histology-based treatment approaches has been provided by clinical trials of targeted and more novel chemotherapy drugs, which have demonstrated that outcomes are dependent on the histological subgroup (8–11). Angiogenesis is particularly critical to tumor growth and metastatic dissemination, and the overexpression of VEGF has been associated with a poor prognosis in patients with NSCLC (12,13).

Bevacizumab is an anti-VEGF monoclonal antibody that has been revealed to inhibit tumor-associated angiogenesis in preclinical and clinical studies (14,15).

In the phase III Eastern Cooperative Oncology Group (ECOG) E4599 and Avastin in Lung trials, the addition of bevacizumab to platinum-based doublet chemotherapy resulted in significant improvements in the overall response rate and median progression-free survival (PFS) time (16,17). Bevacizumab has also been tested in Japanese patients with NSCLC. For example, in the Japanese phase II JO19907 trial, the addition of bevacizumab to paclitaxel and carboplatin resulted in a significant improvement in the median PFS time compared with paclitaxel and carboplatin alone (6.9 vs. 5.9 months; P=0.009) (18).

Although pemetrexed and bevacizumab have each demonstrated efficacy in patients with non-squamous NSCLC, less is known about their effects in combination. In a multicenter phase II trial, the combination of pemetrexed and carboplatin plus bevacizumab followed by maintenance pemetrexed and bevacizumab exhibited encouraging activity in chemotherapy-naïve patients with advanced non-squamous NSCLC (19). The response rate was 55% (95% CI, 41–69), the median PFS time was 7.8 months (95% CI, 5.2–11.5) and the median OS time was 14.1 months (95% CI, 10.8–19.6). The efficacy and safety of this regimen, however, was not assessed specifically in Japanese patients. The present study therefore evaluated the efficacy and safety of pemetrexed and carboplatin plus bevacizumab, followed by maintenance pemetrexed and bevacizumab, in Japanese patients with advanced non-squamous NSCLC.

## Patients and methods

### Patients

The present study consisted of patients who were aged 20–75 years, with stage IIIB, stage IV or recurrent NSCLC, as confirmed histologically or cytologically, and who were naïve to chemotherapy. Each patient had at least one unidimensionally measurable lesion according to the Response Evaluation Criteria in Solid Tumors (20), an ECOG performance status of 0 or 1 (21), and adequate hematological, hepatic and renal functions, including a urine protein/creatinine level of ≤1.0 mg/dl and a creatinine clearance of >45 ml/min.

Exclusion criteria included the following: Histological evidence of a predominantly squamous cell cancer; a primary tumor in close proximity to a major vessel or with cavitation; a history of gross hemoptysis (≥2.5 ml); brain metastases or prior treatment for brain metastasis; uncontrolled pleural or pericardial effusion or ascites; a severe and uncontrolled complication; uncontrollable diabetes mellitus or hypertension (blood pressure, ≥150/100 mmHg); clinically significant cardiovascular disease, including unstable angina pectoris; pregnancy or lactation; a history of thrombotic or hemorrhagic disorder; regular use of aspirin (>325 mg/day); use of non-steroidal anti-inflammatory agents or other agents known to inhibit platelet function; radiation within 21 days of enrollment; major surgery within 28 days of enrollment; history of active double cancer; an unstable psychiatric disorder; or a decision of ineligibility provided by a physician.

The present study (trial no. UMIN000003387) was conducted in accordance with the Declaration of Helsinki and Good Clinical Practice guidelines and approved by the institutional review board of Kansai Medical University (Hirakata, Japan). Patients were required to provide informed consent.

### Treatment plan

The present study was a single-arm phase II trial of first-line pemetrexed, carboplatin and bevacizumab, followed by maintenance pemetrexed and bevacizumab. Eligible patients were administered pemetrexed (500 mg/m^2^), carboplatin (area under the concentration-time curve, 6.0 mg/ml × min) and bevacizumab (15 mg/kg) intravenously every three weeks for four to six cycles, unless there was evidence of disease progression or intolerance to the treatment. Patients who achieved a complete response (CR), partial response (PR) or stable disease (SD) were subsequently administered maintenance therapy, consisting of intravenous pemetrexed (500 mg/m^2^) and bevacizumab (15 mg/kg) every three weeks until there was evidence of disease progression or development of unacceptable toxicities.

All patients received oral folic acid (500 μg/day) and a vitamin B_12_ injection (1,000 μg every nine weeks), beginning one to two weeks prior to the first dose of pemetrexed, carboplatin and bevacizumab, and continuing until three weeks after the last dose of the treatment.

### Endpoints

The primary endpoint of this phase II trial was the response rate (RR). Secondary endpoints included PFS and OS times, time to response and safety.

### Assessment of objective response

Prior to entering the study, a medical history was taken, and then the patients underwent a physical examination, measurements of any palpable lesions and an assessment of lesions by computed tomography. Tumor responses were assessed radiographically every four weeks for 12 weeks and every four to eight weeks thereafter until disease progression occurred. The disease status was assessed according to the Response Evaluation Criteria in Solid Tumors (20) and toxicities were graded according to the National Cancer Institute Common Toxicity Criteria, version 4.0 (22)

### Statistical analyses

The response rate (RR) was set as the primary endpoint of the present study. The RR of platinum-based doublet chemotherapy for Japanese NSCLC patients has been reported to be ~30% (23). Also, the RR of cisplatin plus pemetrexed has been reported to be the same as that of cisplatin plus gemcitabine. In addition, the RR was recorded as 31 and 60.7%, respectively, in a Japanese phase II trial comparing carboplatin plus paclitaxel with carboplatin plus paclitaxel and bevacizumab. Therefore, it was expected that bevacizumab could increase the RR by 30% for the carboplatin plus pemetrexed regimen. Using the SWOG statistical tool (SWOG Statistical Center, Seattle, WA, USA), a total of 23 patients were evaluated to explain the present hypothesis, to disregard a RR of 30% and to provide a two-sided significance level of <0.1, with a statistical power of 90%, to assess the activity of the regimen as a 60% RR. Finally, a target sample size of 25 patients was chosen on the expectation that a proportion of patients would prove to be ineligible for the study. The main analysis of efficacy was conducted on the full analysis set, which was produced by omitting ineligible patients.

The time-to-event variables obtained from the Kaplan-Meier method were determined by log-rank tests. P<0.05 was considered to indicate a statistically significant difference. Statistical analyses were conducted by JMP software (version 9; SAS Institute Inc., Cary, NC, USA).

## Results

### Patient characteristics

Between March 2010 and January 2011, 26 newly diagnosed patients with advanced non-squamous NSCLC were enrolled in the present study at Kansai Medical University Hirakata Hospital (Hirakata, Osaka, Japan). Three patients were excluded, as they were diagnosed with brain metastasis prior to treatment. The ITT population therefore consisted of 23 patients, and their demographic and clinical characteristics are shown in Table I.

### Treatment

The 23 eligible patients received a median of six cycles (range, four to six cycles) of induction chemotherapy, consisting of pemetrexed, carboplatin and bevacizumab. Of these patients, 13 (56.5%) received maintenance chemotherapy, consisting of pemetrexed and bevacizumab, for a median of four cycles (range, 0–27 cycles).

Reasons for discontinuation of induction chemotherapy included disease progression (n=3), unacceptable toxicity (n=3) and patient request (n=4). Reasons for discontinuation of the maintenance chemotherapy were disease progression (n=9) and unacceptable toxicity (n=3); one patient remains on maintenance chemotherapy at the present time.

Seven (30.4%) of the 23 patients had one or more dose reductions, two patients had two reductions each and two patients had more than two dose reductions each.

### Efficacy

**Figure 1:**
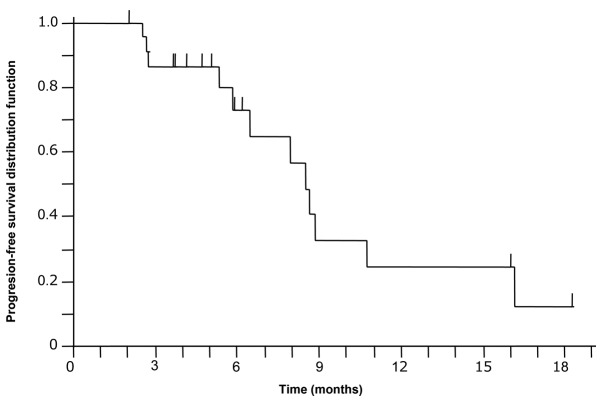
Kaplan-Meier curve for progression-free survival. The median progression-free survival time was 8.6 months (95% confidence interval, 5.9–10.9 months).

**Figure 2:**
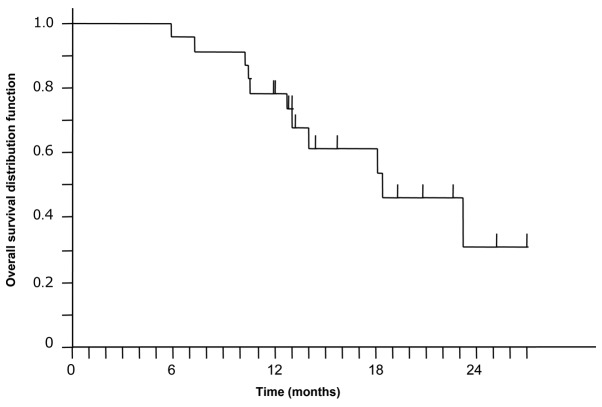
Kaplan-Meier curve for overall survival. The median overall survival time was 18.6 months (95% confidence interval, 12.9–24.8 months).

Of the 23 patients, three (13.0%) achieved a CR and 13 (56.5%) achieved a PR, making the RR 69.6% (95% CI, 47.1–86.8). The other seven patients (30.4%) achieved SD as their best response to therapy, making the disease control rate (DCR) 100% (Table II). The time to response was 1.2 months (95% CI, 0.72–1.93). At a median follow-up of 13.4 months (range, 5.9–27.4), the median PFS time was 8.6 months (95% CI, 5.9–10.9; Fig. 1) and the median OS time was 18.6 months (95% CI, 12.9–24.8 months; Fig. 2).

### Safety

All adverse events (AEs) are listed in Table III. AEs of grade 3 or higher were observed in 15 patients (65.2%). During cycles four to six of induction chemotherapy, the grade 3 and 4 hematological AEs included neutropenia (30.4%), thrombocytopenia (17.4%), leucopenia (13.0%) and anemia (4.3%), and the grade 3 and 4 non-hematologic AEs included liver damage (13.0%), fatigue (8.7%) and proteinuria (8.7%). Toxicity during the maintenance phase was minimal, with one patient each experiencing grade 3 leukocytopenia, anemia, fatigue and proteinuria. No patient experienced grade 3 or greater hypertension or venous thrombosis.

## Discussion

This single institution phase II study revealed that induction therapy with a combination of pemetrexed and carboplatin plus bevacizumab, followed by maintenance therapy with pemetrexed and bevacizumab, was effective and well-tolerated in chemotherapy-naïve Japanese patients with advanced non-squamous NSCLC. The RR was 69.6%, the DCR was 100%, the median PFS time was 8.6 months and the median OS time was 18.6 months. To the best of our knowledge, the present study is the first to evaluate the efficacy of this regimen in chemotherapy-naïve Japanese patients with advanced non-squamous NSCLC.

NSCLC was, until recently, considered to be a single disease, with first-line treatment of platinum-based doublet chemotherapy for patients with advanced disease. Carboplatin plus paclitaxel, the platinum-based doublet chemotherapy most frequently administered to patients with NSCLC, has been shown to exhibit an RR of 17%, a median PFS time of 3.1 months and a median OS time of 8.1 months (24).

Angiogenesis is important for tumor growth and metastasis, with the pro-angiogenic protein, VEGF, being a major regulator of angiogenesis in normal and malignant tissues (11,25,26). The overexpression of VEGF has been correlated with a poor prognosis in patients with NSCLC (12,13). Bevacizumab (Avastin; Genentech, South San Francisco, CA, USA) is a monoclonal antibody against VEGF that can impede tumor-associated angiogenesis. In a phase III randomized controlled trial, bevacizumab plus carboplatin and paclitaxel, followed by maintenance therapy with bevacizumab, was compared with carboplatin and paclitaxel in the treatment of patients with non-squamous NSCLC. The addition of bevacizumab improved the RR (35 vs. 15%), the median PFS time (6.2 vs. 4.5 months) and the median OS time (12.3 vs. 10.3 months) (16). Another phase III trial revealed that the efficacy of cisplatin plus pemetrexed was similar to that of cisplatin plus gemcitabine, a standard platinum-based doublet chemotherapy, but that cisplatin plus pemetrexed resulted in significantly fewer AEs (6). In addition, the OS time was longer with cisplatin plus pemetrexed compared with cisplatin plus gemcitabine in patients with non-squamous NSCLC, including those with adenocarcinoma (12.6 vs. 10.9 months) and large cell carcinoma (10.4 vs. 6.7 months). A more recent phase III randomized trial revealed that maintenance therapy with pemetrexed following first-line induction treatment with pemetrexed and cisplatin improved the PFS time in patients with advanced non-squamous NSCLC (7).

Taken together, these findings suggested that induction chemotherapy with platinum and pemetrexed plus bevacizumab, followed by maintenance pemetrexed and bevacizumab, would be effective in patients with advanced non-squamous NSCLC. Indeed, a phase II trial of carboplatin and pemetrexed plus bevacizumab, followed by maintenance pemetrexed and bevacizumab, exhibited a good RR (55%; 95% CI, 41–69), median PFS time (7.8 months; 95% CI, 5.2–11.5) and median OS time (14.1 months; 95% CI, 10.8–19.6) in Western patients with non-squamous NSCLC (19).

In a phase III trial, bevacizumab plus carboplatin and pemetrexed, followed by maintenance therapy with pemetrexed plus bevacizumab, significantly improved the PFS time in the treatment of patients with non-squamous NSCLC, although the OS time was not significantly improved (27). However, this regimen had not been tested in Japanese patients.

Japanese ethnicity has been reported to be a favorable prognostic factor for OS in NSCLC patients, with a higher RR and longer OS time in Japanese patients compared with Caucasian patients with NSCLC. Patients in the phase III ECOG E4599 trial who were treated with carboplatin and paclitaxel with bevacizumab had an RR of 35% and a median OS time of 12.3 months (16), whereas those in the Japanese phase II JO19907 trial had an RR of 60.7% and a median OS time of 22.8 months (18). These findings suggested that the combination of carboplatin and pemetrexed plus bevacizumab, followed by maintenance pemetrexed and bevacizumab, would be effective and safe in Japanese patients with non-squamous NSCLC. Indeed, it was found in the present study that this regimen was effective in Japanese patients. The observed time to response in the present study was 1.2 months, similar to that observed in the JO19907 trial, suggesting that the addition of bevacizumab to chemotherapy results in rapid tumor size reduction. In addition, tumor-related symptoms were improved in the NSCLC patients who achieved a PR compared with SD following two cycles of first-line chemotherapy, thus improving patient quality of life.

This regimen was also demonstrated to be safe, as grade 4 hematological AEs were observed in only four patients, two each with neutropenia and thrombocytopenia, and grade 4 non-hematological AEs were not observed in any patients. AEs associated with bevacizumab, including hypertension and venous thrombosis, were also not observed.

In conclusion, treatment with induction chemotherapy, consisting of carboplatin and pemetrexed plus bevacizumab, followed by maintenance chemotherapy with pemetrexed and bevacizumab, was effective and tolerable in previously untreated Japanese patients with advanced non-squamous NSCLC, with a relatively short period to response. These results suggest that the regimen described in the present study should be tested in randomized, controlled trials of first-line treatment for Japanese patients with non-squamous NSCLC.

## Figures and Tables

**Table I:** Patient characteristics.

Parameters	Value
Median age years, range	64 (40–74)
Gender, n (%)	
Male	15 (65.2)
Female	8 (34.8)
ECOG PS, n (%)	
0	6 (26.1)
1	17 (73.9)
Disease stage, n (%)	
IIIB	3 (13.0)
IV	20 (87.0)
Histology, n (%)	
Adenocarcinoma	23 (100.0)
EGFR, n (%)	
Mutant	3 (13.0)
Wild-type	16 (69.6)
Unknown	4 (17.4)

EGFR, epidermal growth factor receptor; ECOG, Eastern Cooperative Oncology Group; PS, performance status.

**Table II:** Response (n=23).

Parameters	n (%)
CR	3 (13.0)
PR	13 (56.5)
SD	7 (30.4)
Progressive disease	0 (0.0)
Response rate (CR+PR)	16 (69.6)
Disease control rate (CR+PR+SD)	23 (100.0)

CR, complete response; PR, partial response; SD, stable disease.

**Table III:** Toxicities.

Adverse events	Any grade, n (%)	Grade 3, n (%)	Grade 4, n (%)
Leukocytopenia	12 (52.2)	3 (13.0)	0 (0.0)
Neutropenia	11 (47.8)	5 (21.7)	2 (8.7)
Thrombocytopenia	16 (69.6)	2 (8.7)	2 (8.7)
Anemia	15 (65.2)	1 (4.3)	0 (0.0)
Hypoalbuminemia	7 (30.4)	0 (0.0)	0 (0.0)
AST increased	16 (69.6)	2 (8.7)	0 (0.0)
ALT increased	17 (73.9)	3 (13.0)	0 (0.0)
Creatinine increased	2 (8.7)	0 (0.0)	0 (0.0)
Blood bilirubin increased	4 (17.4)	0 (0.0)	0 (0.0)
Blood K increased	2 (8.7)	0 (0.0)	0 (0.0)
Fatigue	11 (47.8)	2 (8.7)	0 (0.0)
Nausea	12 (52.2)	0 (0.0)	0 (0.0)
Vomiting	4 (17.4)	0 (0.0)	0 (0.0)
Anorexia	14 (60.9)	0 (0.0)	0 (0.0)
Skin	1 (4.3)	0 (0.0)	0 (0.0)
Hypertension	3 (13.0)	0 (0.0)	0 (0.0)
Proteinuria	11 (47.8)	2 (8.7)	0 (0.0)
Hemoptysis	2 (8.7)	0 (0.0)	0 (0.0)
Venous thrombosis	1 (4.3)	0 (0.0)	0 (0.0)
Mucositis oral	4 (17.4)	0 (0.0)	0 (0.0)
Fever without neutropenia	4 (17.4)	0 (0.0)	0 (0.0)
Alopesia	4 (17.4)	0 (0.0)	0 (0.0)
Peripheral neuropathy	4 (17.4)	0 (0.0)	0 (0.0)
Dysgeusia	1 (4.3)	0 (0.0)	0 (0.0)
Pneumonitis	1 (4.3)	0 (0.0)	0 (0.0)
Diarrhea	3 (13.0)	0 (0.0)	0 (0.0)
Constipation	9 (39.1)	0 (0.0)	0 (0.0)

AST, aspartate aminotransferase; ALT, alanine transaminase.
